# Presence of the narrow-spectrum OXA-1 β-lactamase enzyme is associated with elevated piperacillin/tazobactam MIC values among ESBL-producing *Escherichia coli* clinical isolates (CANWARD, 2007–18)

**DOI:** 10.1093/jacamr/dlac027

**Published:** 2022-03-21

**Authors:** A. Walkty, J. A. Karlowsky, P. R. S. Lagacé-Wiens, A. R. Golden, M. R. Baxter, A. J. Denisuik, M. McCracken, M. R. Mulvey, H. J. Adam, G. G. Zhanel

**Affiliations:** Department of Medical Microbiology and Infectious Diseases, Max Rady College of Medicine, University of Manitoba, Winnipeg, Canada; Shared Health, Winnipeg, Canada; Department of Medical Microbiology and Infectious Diseases, Max Rady College of Medicine, University of Manitoba, Winnipeg, Canada; Shared Health, Winnipeg, Canada; Department of Medical Microbiology and Infectious Diseases, Max Rady College of Medicine, University of Manitoba, Winnipeg, Canada; Shared Health, Winnipeg, Canada; Public Health Agency of Canada – National Microbiology Laboratory, Winnipeg, Canada; Department of Medical Microbiology and Infectious Diseases, Max Rady College of Medicine, University of Manitoba, Winnipeg, Canada; Department of Medical Microbiology and Infectious Diseases, Max Rady College of Medicine, University of Manitoba, Winnipeg, Canada; Public Health Agency of Canada – National Microbiology Laboratory, Winnipeg, Canada; Public Health Agency of Canada – National Microbiology Laboratory, Winnipeg, Canada; Department of Medical Microbiology and Infectious Diseases, Max Rady College of Medicine, University of Manitoba, Winnipeg, Canada; Shared Health, Winnipeg, Canada; Department of Medical Microbiology and Infectious Diseases, Max Rady College of Medicine, University of Manitoba, Winnipeg, Canada

Piperacillin/tazobactam has been proposed as an alternative to carbapenems for the management of infections caused by ESBL-producing *Escherichia coli*.^1^ A recent randomized, controlled trial (MERINO study) that compared piperacillin/tazobactam to meropenem for the treatment of bacteraemia due to ceftriaxone non-susceptible *E. coli* and *Klebsiella pneumoniae* found an increase in 30 day mortality in the piperacillin/tazobactam arm.^2^ However, data from this trial and others suggest that the clinical outcome among patients receiving piperacillin/tazobactam appears to depend, at least in part, on the MIC for this antimicrobial.^3^ The purpose of this study was to explore the association between the MIC of piperacillin/tazobactam and the presence of OXA-1 among ESBL-producing *E. coli* using a collection of isolates obtained from an ongoing national surveillance study in Canada (CANWARD).

*E. coli* clinical isolates were obtained from patients admitted to or evaluated at sentinel hospitals across Canada (January 2007 to December 2018) as part of an ongoing national surveillance study (CANWARD).^4^ On an annual basis, each centre was asked to submit clinical isolates (consecutive, one per patient/infection site) from blood, respiratory, urine and wound infections. Isolate identification was performed by the submitting site and confirmed at the reference site as required. Isolates were shipped on Amies semi-solid transport media to the coordinating laboratory (Health Sciences Centre, Winnipeg, Canada), subcultured onto appropriate media and stocked in skim milk at −80°C until MIC testing was carried out.

Following two subcultures from frozen stock, the *in vitro* activity of piperacillin/tazobactam was determined by broth microdilution in accordance with the CLSI standards.^5^ In-house-prepared 96-well broth microdilution panels were used for antimicrobial susceptibility testing. Putative ESBL-producing *E. coli* were identified as isolates with a ceftriaxone and/or ceftazidime MIC of ≥1 mg/L. ESBL production was verified using the CLSI phenotypic confirmatory disc test.^6^

All phenotypically confirmed ESBL-producing isolates were sequenced using the Illumina MiSeq platform. Quality control was performed using the FastQC tool (http://www.bioinformatics.babraham.ac.uk/projects/fastqc/) and contigs were assembled using SPAdes software.^7^ β-lactamase genes were identified using ResFinder 4.0 at an identity threshold of 90%.^8^ MLST alleles and STs were identified by scanning assembled contigs against available PubMLST databases (https://github.com/tseemann/mlst).

In total, 671 ESBL-producing *E. coli* were identified as part of the CANWARD study, of which 62.4% (419/671) were ST131. The majority of isolates (92.0%; 617/671) harboured a CTX-M ESBL enzyme. CTX-M-15 (62.3%; 418/671), CTX-M-27 (13.9%; 93/671) and CTX-M-14 (13.4%; 90/671) were the most common variants identified. The narrow spectrum OXA-1 β-lactamase enzyme was present in 42.6% (286/671) of isolates. OXA-1 was detected in 66.3% (277/418) of isolates with a CTX-M-15 ESBL enzyme versus only 3.6% (9/253) of isolates with other ESBL enzyme types. The piperacillin/tazobactam MIC_50_ and MIC_90_ values were 8 mg/L and 32 mg/L for isolates that possessed the OXA-1 enzyme (modal MIC of 8 mg/L) versus 2 mg/L and 8 mg/L for those that did not (modal MIC of mg/L). The percentage of ESBL-producing *E. coli* that were inhibited by a piperacillin/tazobactam MIC of ≤8 mg/L (EUCAST susceptibility breakpoint)^9^ was 68.5% for isolates that were OXA-1 positive and 93.8% for isolates that were OXA-1 negative. Presence (*n *= 195) or absence (*n *= 476) of the narrow-spectrum TEM-1 enzyme did not appear to influence the association of OXA-1 with elevated MICs for piperacillin/tazobactam (Table 1).

**Table 1.** Piperacillin/tazobactam MIC distribution for ESBL-producing *E. coli* clinical isolates, stratified by the presence or absence of OXA-1

Organism (subset)	Piperacillin/tazobactam MIC (mg/L)										Total
	≤1	2	4	8	16	32	64	128	256	≥512	
ESBL-producing *E. coli* (all)											671
OXA-1 absent (*n *= 385)	107 (27.8)	148 (66.2)	88 (89.1)	18 (93.8)	10 (96.4)	4 (97.4)	2 (97.9)	0 (97.9)	5 (99.2)	3 (100.0)	385
OXA-1 present (*n *= 286)	10 (3.5)	27 (12.9)	66 (36.0)	93 (68.5)	55 (87.8)	7 (90.2)	15 (95.5)	7 (97.9)	2 (98.6)	4 (100.0)	286
ESBL-producing *E. coli* (CTX-M-15-positive subset)											418
OXA-1 absent (*n *= 141)	31 (22.0)	57 (62.4)	38 (89.4)	7 (94.3)	3 (96.5)	0 (96.5)	1 (97.2)	0 (97.2)	2 (98.6)	2 (100.0)	141
OXA-1 present (*n *= 277)	9 (3.2)	27 (13.0)	65 (36.5)	87 (67.9)	55 (87.8)	7 (90.3)	15 (95.7)	6 (97.8)	2 (98.6)	4 (100.0)	277
ESBL-producing *E. coli* (CTX-M-15-negative subset)											253
OXA-1 absent (*n *= 244)	76 (31.1)	91 (68.4)	50 (88.9)	11 (93.4)	7 (96.3)	4 (98.0)	1 (98.4)	0 (98.4)	3 (99.6)	1 (100.0)	244
OXA-1 present (*n *= 9)	1 (11.1)	0 (11.1)	1 (22.2)	6 (88.9)	0 (88.9)	0 (88.9)	0 (88.9)	1 (100.0)			9
ESBL-producing *E. coli* (TEM-1-positive subset)											194
OXA-1 absent (*n *= 138)	31 (22.3)	55 (61.9)	39 (89.9)	6 (94.2)	3 (97.1)	2 (98.6)	0 (98.6)	0 (98.6)	1 (99.3)	1 (100.0)	138
OXA-1 present (*n *= 56)	4 (7.1)	9 (23.3)	7 (35.7)	14 (60.7)	15 (87.5)	1 (89.3)	3 (95.6)	1 (96.4)	1 (98.2)	1 (100.0)	56
ESBL-producing *E. coli* (TEM-1-negative subset)											477
OXA-1 absent (*n *= 247)	76 (30.9)	93 (68.7)	49 (88.6)	12 (93.5)	7 (96.0)	2 (96.8)	2 (97.6)	0 (97.6)	4 (99.2)	2 (100.0)	247
OXA-1 present (*n *= 230)	6 (2.6)	18 (10.4)	59 (36.1)	79 (70.4)	40 (87.8)	6 (90.4)	12 (95.7)	6 (98.3)	1 (98.7)	3 (100.0)	230

Data are shown as number of isolates (cumulative percentage of isolates).

Henderson *et al.*^3^ recently investigated whether there was any association between the MIC of piperacillin/tazobactam and the presence of specific β-lactamase genes among isolates obtained from patients who participated in the MERINO trial. Of 143 ST131 *E. coli*, the modal piperacillin/tazobactam MIC was 8 mg/L for isolates harbouring CTX-M-15 and OXA-1 versus 2 mg/L for isolates harbouring only CTX-M-15.^3^ Livermore *et al.*^10^ assessed whether the presence of OXA-1 was associated with a reduction in piperacillin/tazobactam susceptibility among 293 ESBL-producing *E. coli* recovered from a bloodstream source of infection. Similar to our dataset, CTX-M-15 was the most common ESBL enzyme identified (present in 78.2% of isolates). The modal MIC for piperacillin/tazobactam was 8 or 16 mg/L (depending on the subgroup evaluated) for ESBL producers in the presence of OXA-1 versus 2 mg/L for ESBL producers in the absence of OXA-1.^10^ The results from these studies are largely in keeping with the data presented in this report. The association of OXA-1 with CTX-M-15 described here and elsewhere is particularly concerning given the widespread distribution of CTX-M ESBL enzymes worldwide.^1,3,10^

In summary, among a large collection of ESBL-producing *E. coli* clinical isolates obtained from patients at Canadian hospitals, the MIC_50_, MIC_90_ and modal MIC values of piperacillin/tazobactam were higher for the subset that harboured a narrow-spectrum OXA-1 β-lactamase enzyme relative to the subset that did not. The most important limitation of our study is that OXA-1 was infrequently detected in ESBL-producing *E. coli* that did not contain CTX-M-15. As many clinical microbiology laboratories use automated instruments for determination of piperacillin/tazobactam susceptibility, absence of OXA-1 by molecular testing may prove useful in further defining a subset of piperacillin/tazobactam susceptible ESBL-producing *E. coli* for which therapeutic use of this antimicrobial is most appropriate.
